# The Use of the Muscles of the Uvula in Deglutition

**Published:** 1868-09

**Authors:** Thomas Brian Gunning


### AMERICAN JOURNAL

OF

### DENTAL SCIENCE.

**Vol. 11. THIRD SERIES--SEPTEMBER, 1868. No. 5.**

### ARTICLE I.

### *The Use of the Muscles of the Uvula in Deglutition.*

By Thomas Brian Gunning.

My object at this time, is to show the most important use or function, of the muscles of the Uvula; not to explain the mechanism of deglutition fully.

The opinions of physiologists have differed materially in regard to the use of these muscles in deglutition, but no correct or satisfactory explanation has been given of them by any one. The following quotation may be taken as fairly representing what is now believed of them. "The Azygos Uvulæ is the small muscle, consisting of two fasciculi, one on either side, which forms the fleshy portion of the uvula. It has no very marked or important function in deglutition." *

My own views are as follows:

When the food has passed into the upper part of the pharynx, it is shut in by a band or welt, consisting of the forward portion of the soft palate, continued down the sides, by the anterior pillars. The upper portion, is formed by the action of the Tensores Palati muscles drawing their aponeuroses

* The Physiology of Man, by Austin Flint M. D. Vol. 2nd. page 185. New York, 1867.

tight, and the Palato-glossi coming into action, and continuing the curve down, on each side of the tongue, at the same time assisting to draw the latter up against this arched band, or welt, by which the food is kept back. It should be understood that the upper part of this arch is formed by the aponeurosis, at some distance in *front* of the uvula, so that the part of the soft palate behind the welt, is left free. Through the middle of this, the Azygos Uvulæ muscles pass to the uvula, in the centre of the back border, or arch, formed by the *Palato-Pharyngeus* curving down on each side, and known as the posterior pillars of the soft palate. These two pairs of muscles, are inactive, as the *Levatores Palati* have drawn the soft palate up behind, and closed the passage to the posterior nares, while the food is shut in at the front, as before described. At the instant this is accomplished, the Palato-pharyngei act, and come together behind; the Levatores palati relax; and the *Azygos Uvulæ* muscles come strongly into action, and draw the uvula, and the origins of the Palato-pharyngei rapidly forward. The Azygos Uvulae muscles, which pass from the spine of the hard palate, to the uvula, are at this time held *down* to the tongue, by the welt or band formed by the aponeurosis before mentioned, consequently, they now in acting, draw the origins of the Palato Pharyngei down to, and forward along the upper surface of the tongue; and as the insertions of these muscles extend down around the sides and back of the pharynx (crossing each other behind) they, in acting at this time, form a circular layer of muscular fibres which converge from the circumference of the sides, and back of the pharynx, across to the insertion of the Azygos Uvulae muscles. In other words a layer of muscular fibres spreads out, from each side, meets in the centre, behind the food, and the point to which they converge, being drawn down to, and forward on a level with the upper surface of the tongue, the food is pressed down the pharynx. Again, by this arrangement of the parts, a muscular arch draws down close against the upper surface of the tongue, to keep the food back; while the remainder of the soft palate shuts off the posterior nares. This last then changes into a muscular curtain, behind, and then into a roof *over* the food, and as the circumference of this roof, is down on the face of the middle and inferior constrictor muscles, and the origins of the Azygos Uvulæ muscles is on the apex of the spine of the hard palate, there is a plane of muscles, contracting over the food, and coming down to the line of their attachment behind, and the level of the tongue in front. If the finger is placed along the tongue, until its end touches the centre of the welt formed to keep the food back in the pharynx, the Azygos Uvulæ muscles may be felt tightening, as the saliva is swallowed; and if the end of the finger is passed forward under the welt; on swallowing, the uvula will pass forward, and turn up, so much, that its back will be felt, against the back of the finger, if the latter is held just low enough. The *Azygos Uvulæ* muscles always act in deglutition, whether solid or liquid food, or merely the saliva is swallowed. It is thus shown that the Tensores Palati muscles and the Palato-glossi act in concert, to form the arched band which shuts down against the tongue, and that the Palato-pharyngei are not associated with the Palato-glossi in constricting the isthmus of the fauces. The Palato-pharyngei, however, act in concert with the Azygos Uvulæ, to press the food down the pharynx. In doing this the Azygos Uvulae (in converting the screen, or curtain formed by the closing of the Pharyngei behind the food, into a roof or cover, by drawing their origins at the uvula downward and forward,) make the Pharyngei muscles act as if they were prolonged to the spine of the hard palate. Another important feature, is that the Azygos Uvulae muscles are the direct antagonists of the Levatores-Palati. The influence of this antagonism will not, however, be dwelt upon at this time.

The foregoing explanations show, that, every muscle of the soft palate, is active, in deglutition, and that the food is effectually controlled without unreasonable action on the part of any muscle such as that generally imputed to the

Superior Constrictor, which cannot act in deglutition, as supposed, its attachments making it impossible that it can-press the food down the pharynx.

